# An ISAR Image Component Recognition Method Based on Semantic Segmentation and Mask Matching

**DOI:** 10.3390/s23187955

**Published:** 2023-09-18

**Authors:** Xinli Zhu, Yasheng Zhang, Wang Lu, Yuqiang Fang, Jun He

**Affiliations:** 1 Graduate School, Space Engineering University, Beijing 101416, China; zhuxinli2021@163.com (X.Z.); hj1997@stu.xjtu.edu.cn (J.H.); 2 Department of Aerospace Science and Technology, Space Engineering University, Beijing 101416, China; zhangyspublic@163.com (Y.Z.); fangyuqiang@nudt.edu.cn (Y.F.); 3 Beijing Institute of Tracking and Telecommunication Technology, Beijing 100094, China

**Keywords:** inverse synthetic aperture radar (ISAR), component recognition, semantic segmentation, U-Net, Siamese network

## Abstract

The inverse synthetic aperture radar (ISAR) image is a kind of target feature data acquired by radar for moving targets, which can reflect the shape, structure, and motion information of the target, and has attracted a great deal of attention from the radar automatic target recognition (RATR) community. The identification of ISAR image components in radar satellite identification missions has not been carried out in related research, and the relevant segmentation methods of optical images applied to the research of semantic segmentation of ISAR images do not achieve ideal segmentation results. To address this problem, this paper proposes an ISAR image part recognition method based on semantic segmentation and mask matching. Furthermore, a reliable automatic ISAR image component labeling method is designed, and the satellite target component labeling ISAR image samples are obtained accurately and efficiently, and the satellite target component labeling ISAR image data set is obtained. On this basis, an ISAR image component recognition method based on semantic segmentation and mask matching is proposed in this paper. U-Net and Siamese Network are designed to complete the ISAR image binary semantic segmentation and binary mask matching, respectively. The component label of the ISAR image is predicted by the mask matching results. Experiments based on satellite component labeling ISAR image datasets confirm that the proposed method is feasible and effective, and it has greater comparative advantages compared to other classical semantic segmentation networks.

## 1. Introduction

Space target recognition is the main function of space surveillance information systems, where satellites in orbit play an important role and are key targets for recognition. However, there are few published research results in this area. The difficulty of this problem is that satellites are too small or too far away to identify detailed information, and it is relatively difficult to obtain effective identification data. Compared with other methods of obtaining target characteristic information, radar, as a radio detection device, has inherent advantages such as all-weather, real-time, and long detection range. These advantages make it an important sensor to realize the automatic identification of sea, air, and sky targets. Moreover, with the rapid development of modern radar technology, the rapid emergence of broadband radar has greatly expanded the information acquisition capability of radar systems for targets and environments, making broadband radar an important means to obtain target characteristic data. These data mainly include high-resolution range images (HRRP), inverse synthetic aperture radar (ISAR) images, and synthetic aperture radar (SAR) images, which are widely used in radar automatic target recognition (RATR) research [1,2]. In satellite target recognition studies, HRRP and ISAR images are commonly used as target characterization data; HRRP is a one-dimensional projection of the target scattering center along the radar line of sight (LOS), and ISAR images achieve two-dimensional high-resolution imaging of moving targets by obtaining high resolution in the distance and azimuth directions. Compared with HRRP, ISAR images can provide rich and distinguishable shape, structure, and motion information, which makes ISAR images automatic target recognition research highly interesting in the RATR community [3,4,5].

Research on satellite target recognition technology based on ISAR images has been carried out, and deep learning and sample-less learning methods have been used to identify the specific model of satellite targets, and more satisfactory results have been achieved [6,7]. The research of satellite target component identification technology based on ISAR images can be realized because it is possible to identify not only the target category type but also the target component level using 2D high-resolution ISAR images [8,9,10]. There are some difficulties in the study of ISAR image component recognition. Compared with optical images, ISAR images generally have no complex background, and the information available for feature extraction is usually only intensity, phase, polarization scattering, etc., and there is no available information such as optical image color, texture, etc. Moreover, the backward radiation method also leads to different amplitude values of the same component in different ISAR images, and the signal intensity changes dynamically, which makes it difficult for the classical image semantic segmentation model to mine the amplitude pattern of each component in ISAR images of the satellite target [11,12]. Therefore, the segmentation performance is not satisfactory when the relevant segmentation methods of optical images are used to carry out the semantic segmentation study of ISAR images [13]. At the same time, as a relatively cutting-edge research topic in RATR, the ISAR image segmentation methods are rarely researched, and the recognition effect is unsatisfactory [14,15].

To solve the above challenges, this paper adopts the designed annotation method to realize the automatic annotation of satellite target ISAR image components, generates a large number of component annotated ISAR image samples for satellite target simulation, and then constitutes the corresponding component annotated ISAR image dataset, which lays the data foundation for the subsequent research of satellite target component recognition technology based on ISAR images. Then, based on the characteristics of ISAR image data and the difficulties of component recognition, a method of ISAR image component recognition based on semantic segmentation and mask matching is proposed. Finally, the proposed network is trained and tested on the component labeled ISAR image dataset to improve the accuracy and generalization ability of the network, to realize the component recognition of satellite target ISAR images. Its features are summarized as follows:

(1) An ISAR images component recognition method based on semantic segmentation and mask matching is proposed, which uses U-Net and Siamese network to realize ISAR images’ binary semantic segmentation and binary mask matching for the first time, respectively, and the mask matching result is used to predict ISAR images’ component labels. It is confirmed by experiments that the proposed method is feasible and effective for ISAR image component recognition and has significant advantages over classical image semantic segmentation models.

(2) Designed a reliable automatic ISAR image component labeling method to obtain accurate and efficient satellite target component labeling ISAR image samples, and a satellite target component labeled ISAR image dataset is obtained with the difficulty of ISAR image component recognition as a theoretical guide.

The rest of this paper is organized as follows: in Section 2, we analyze the work related to semantic segmentation. In Section 3, we construct semantic segmentation and mask-matching structures based on the U-Net network and Siamese network and give the general flowchart of this component identification method. In Section 4, we perform the preparation of the satellite target ISAR images component annotation data, then train the U-Net model and the Siamese model, and finally compare and analyze the performance under different recognition methods and conditions. In Section 5, some conclusions are drawn.

## 2. Related Works

In the field of RATR, there are basically no relevant literature reports on the study of semantic segmentation of ISAR images, and the research at this stage is still mainly focused on ISAR target recognition. However, in the field of optical image and SAR remote sensing, research on semantic segmentation is still a popular area of study, and there is a large amount of literature related to the research of target classification and localization [16], target detection [17], semantic segmentation [18,19], and instance segmentation [20]. To some extent, ISAR image component recognition can be considered as a kind of ISAR image semantic segmentation research.

### 2.1. Traditional Image Semantic Segmentation Methods

Before deep neural networks were proposed, feature extraction and classification algorithm design were two of the most important topics in traditional semantic segmentation research. Scholars proposed or adopted many features for semantic segmentation research, such as pixel color, directional gradient histogram [21], scale-invariant feature transform [22], local binary patterns [23], etc. Semantic segmentation methods also include unsupervised and supervised categories, the simplest of which are thresholding methods for gray images [24], K-means clustering, compression-based methods [25], etc. Traditional semantic segmentation methods are more limited by the extraction of features, and the accuracy is difficult to improve significantly; at the same time, when there are many pixel classes, it is easy to cause problems of computational complexity and training difficulty.

### 2.2. Deep Learning-Based Image Semantic Segmentation Method

Since the rise of deep neural networks, especially the proposed CNN-based Fully Convolutional Network (FCN) [26], the accuracy of image semantic segmentation has been greatly improved. FCN uses all convolutional layers without connection layers and outputs segmentation results with the same size as the input image using an upsampling operation, which eliminates the negative impact of the traditional CNN with all-connected layers on semantic segmentation. Since FCN was proposed, scholars have also designed and proposed many other deep semantic segmentation network models. Considering the large number and complexity of semantic segmentation networks, this paper mainly focuses on the classical network structures and the key techniques in semantic segmentation research. Therefore, the semantic segmentation methods are broadly classified into encoder–decoder-based methods and methods based on integrating contextual information and summarized as follows.

#### 2.2.1. Encoder–Decoder-Based Approach

This class of methods uses an encoder structure consisting of convolution and pooling operations to extract the image feature mapping, and then uses a decoder structure consisting of upsampling to recover the extracted feature mapping to the initial spatial dimension of the input image. Typical networks of this class of methods include SegNet [27], U-Net [28], RefineNet [29], DeepLab series [30,31,32], etc. SegNet obtains feature mappings using encoders and also records the spatial location of the pooling layer maxima in the input image, making it possible to use decoders when U-Net uses shortcut connections to combine the feature mapping information of the encoder and decoder to improve the decoder’s ability to recover the local details of the image, which has achieved satisfactory results in biomedical image segmentation tasks. The encoder of RefineNet is based on the ResNet module, and the decoder is composed of RefineNet blocks, where each block structure contains three parts: residual convolution unit, multiscale fusion, and chained residual pooling, and connects and fuses the high-resolution feature mapping obtained from the encoder with the low-resolution feature mapping from the previous RefineNet blocks. The DeepLab series of methods improve on the shortcomings of FCN and improve the spatial accuracy of semantic segmentation results by obtaining multi-scale image information through several techniques.

#### 2.2.2. Methods Based on Integration of Contextual Information

The basic idea of these methods is to integrate feature mappings at different scales and to find the optimal balance between local and global information. Existing studies have shown that to improve the recognition accuracy of semantic segmentation, both the integration of feature mappings at different scales and the balance between local and global information are required. In semantic segmentation research, using local information is beneficial to improve the classification accuracy at the pixel level, while introducing global information is a key way to solve the local ambiguity problem. Among them, typical methods include conditional random field, convolution with holes, multi-scale feature fusion, etc., which are described as follows:

1. In semantic segmentation research, conditional random field is usually executed as a post-processing step, which takes into account the underlying information of the image (such as inter-pixel relationships) when generating pixel labels, which is very beneficial to the local detail optimization of segmentation results; DeepLab v1 [31], DeepLab v2 [30] use the fully connected conditional random field model as an independent back-end processing step to semantic segmentation results with local detail optimization, achieving better segmentation results. Zheng et al. [33] integrated conditional random field operations into the network model to constitute an end-to-end trained semantic segmentation network model.
2. Convolution with holes, also known as null convolution, is introduced into CNNs to increase the CNN perceptual field exponentially without introducing additional parameters. The advantage of convolution with holes is that it can obtain multi-scale image information, which also makes it unnecessary for CNN to use large-scale pooling operations to expand the perceptual field, thus avoiding the problem of image fine-grained information loss caused by multiple pooling operations. The classical semantic segmentation network models applying convolution with holes include the DeepLab series [30,31,32] and Dilation10 [34].
3. The basic idea of multi-scale feature fusion is that by fusing features at different scales, the feature information of the target can be learned more effectively, which in turn is beneficial to further improve semantic segmentation accuracy. Typical multi-scale feature fusion methods contain atrous spatial pyramid pooling (ASPP), cascade structure, etc. Based on DeepLab v1 convolution with holes, DeepLab v2 [30] and DeepLab v3 [32] further designed and proposed ASPP structures, which use convolution with holes with different expansion rates to extract feature mappings at different scales and fuse them using ASPP structures to improve the semantic segmentation accuracy; PSPNet [35] and RefineNet [29] use a cascade structure to capture feature information at different scales and fuse them.

### 2.3. Loss Function

In image semantic segmentation tasks, scholars have proposed several kinds of semantic segmentation loss functions for different task situations, which play an important role in improving semantic segmentation performance. In the following, several commonly used semantic segmentation loss functions are briefly introduced to lay the foundation for the subsequent experiments of satellite target ISAR image component recognition.

1. Cross-Entropy Loss

Cross-entropy loss is usually used to measure the difference in the probability distribution of a given random variable or event and is widely used in classification tasks, including semantic segmentation tasks for pixel-level classification. It is used as a measure of the distance between the probability distributions $\hat{P}$ and P by adding the KL scatter [36], and the expression is shown in Equation (1). This allows the classifier to quantify the deviation between the predicted probability distribution $\hat{P}$ and the original distribution P for each category i.

(1) $$CE=-\sum_{i}P_{i}\log{\hat{P}}_{i}$$

2. Weighted Cross-Entropy Loss

When the image pixels contain an unbalanced proportion of categories, the classifier tends to focus more on the category with the largest number of samples, making the classification performance biased towards a specific category. In semantic segmentation studies, the number of pixels in various categories is likely to be very different, and this category imbalance is a frequent occurrence. To solve the above problem, it is common practice to utilize the weighted cross-entropy loss, which is a variant of the standard cross-entropy loss with an additional category weight parameter $w_{i}$ and can be expressed as follows:

(2) $$WCE=-\sum_{i}w_{i}P_{i}\log{\hat{P}}_{i}$$

where $w_{i}$ is inversely proportional to the number of pixels in category i.

3. Dice loss

In the field of computer vision, the Dice coefficient is widely used to calculate the similarity of two images, and then it is further improved into a loss function [37], whose expression is as follows:

(3) $$D\left(P,\hat{P}\right)=1-\frac{2P\hat{P}+1}{P+\hat{P}+1}$$

where P is the segmented image true label, while $\hat{P}$ is the segmented image predicted label. The addition of 1 to both numerator and denominator is to ensure that the above loss function is still defined in some special cases (e.g., $P_{i}={\hat{P}}_{i}=0$).

4. Focal Loss

Focal loss is a new variant of weighed cross-entropy loss, as shown in Equation (4), whose important feature is to construct the weight coefficients as a function of the network prediction confidence. By doing this, the difficult-to-predict categories will obtain higher losses than the easy-to-predict categories.

(4) $$FL=-{\sum_{i}\alpha_{i}\left(1-{\hat{P}}_{i}\right)}^{\gamma }\log{\hat{P}}_{i}$$

It uses two additional scaling factors, −α and γ, to control the weights. When γ=0, the focal loss is transformed into the weighted cross-entropy loss, where α is the category weighting coefficient; when γ≥1, the weighting coefficient ${\left(1-{\hat{P}}_{i}\right)}^{\gamma }$ increases as ${\hat{P}}_{i}$ increases, and conversely, when γ<1, the weighting coefficient ${\left(1-{\hat{P}}_{i}\right)}^{\gamma }$ decreases as ${\hat{P}}_{i}$ increases. Therefore, when the ${\hat{P}}_{i}$ of a category is relatively small, its weight coefficient becomes larger, thus increasing the contribution of the category to the loss function. To avoid excessive weighting coefficients, the scaling factor −α can be used to reduce the weights.

5. Lovasz–Softmax Loss

In image semantic segmentation tasks, the Jaccard metric (IoU) is usually used to evaluate image segmentation results. Compared with pixel accuracy (PA), the IoU metric gives proper attention to small targets and counts false negatives (FN) correctly, which is a more suitable semantic segmentation evaluation metric. For this reason, scholars considered optimizing the IoU loss directly during the network training process and designed and proposed a new semantic segmentation loss function, namely Lovasz–Softmax loss [38]. Assuming that p and $\hat{p}$ are the true label and predicted label vectors, respectively, the Jaccard metric for category c is defined as follows:

(5) $$J_{c}\left(p,\hat{p}\right)=\frac{\left|\left\{p=c\right\}\cap \left\{\hat{p}=c\right\}\right|}{\left|\left\{p=c\right\}\cup \left\{\hat{p}=c\right\}\right|}$$

This metric gives the ratio of the intersection of the set of true and predicted labels to their concurrent sets and has a value in the range [0,1]. Then, the corresponding loss function applied in the empirical risk minimization process is

(6) $$\Delta_{J_{c}}\left(p,\hat{p}\right)=1-J_{c}\left(p,\hat{p}\right)$$

In addition, for submodular functions such as Jaccard loss, they further propose to use Lovasz expansion to efficiently minimize the loss function.

## 3. Methods

**Figure 1:**
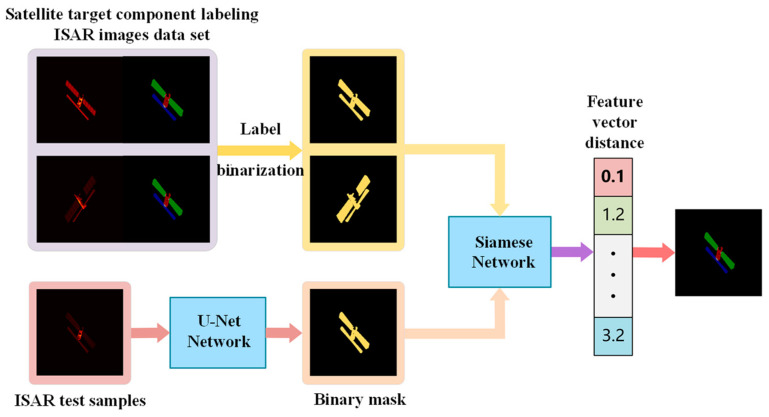
Schematic diagram of the ISAR images component recognition method based on semantic segmentation and mask matching.

In this study, we propose a semantic segmentation and mask-matching-based ISAR image component recognition method, which is shown schematically in Figure 1. The method includes two network models: ISAR image binary semantic segmentation network—U-Net and binary mask matching network—Siamese Network, where the U-Net semantic segmentation network is mainly used to output a binary mask of ISAR images test samples, i.e., to distinguish between ISAR images satellite targets and ISAR image backgrounds. The Siamese network takes the binary masks of the ISAR image data set samples and the binary masks of the ISAR images test samples as input, outputs the distance between the binary masks of the ISAR images test samples and the feature vectors of the binary masks of each ISAR images sample in the data set, and determines the best match of the masks based on the distance size, and then uses the best match of the ISAR images component labels. The component labels of the best-matched ISAR images are then used as the component labels of the test ISAR image samples to achieve satellite target ISAR image component identification. The designed ISAR images semantic segmentation network model and the mask matching network model are described in detail below, respectively.

### 3.1. Semantic Segmentation

Currently, scholars generally adopt deep learning methods to carry out optical image semantic segmentation research, and various network structures have been designed and proposed to implement image semantic segmentation. Among these successfully applied semantic segmentation network models, the fully convolutional-based U-Net [28] uses a symmetric U-shaped structure with compressed and extended paths and achieves better results in the single-channel medical image segmentation task. Therefore, for the single-channel ISAR image component recognition task, U-Net will be used in this study to implement the satellite target ISAR image semantic segmentation; it should be noted that this subsection first only considers the preliminary segmentation recognition of the ISAR image target and background, while the recognition of satellite target ISAR images satellite individual components is not considered for the time being.

**Figure 2:**
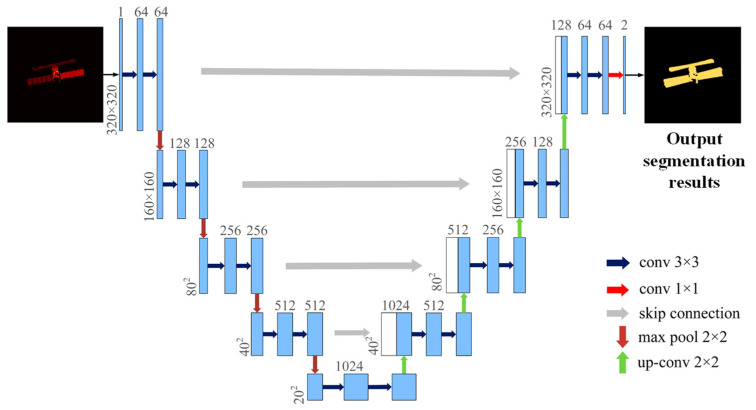
ISAR image binary classification semantic segmentation U-Net network structure.

The network structure of U-Net is shown in Figure 2, which takes a single-channel ISAR image as input and outputs the corresponding binary classification semantic segmentation results. The left side of the network is a series of downsampling operations consisting of convolution and max pool, which consists of four modules, each using two identical convolutions of filter size 3×3 and one max pool operation of filter size 2×2, so that the image size of the ISAR image is reduced to half of the original size after each module. In addition, the number of channels of the feature mapping is doubled after each downsampling, so that the size of the ISAR image with the original size is changed to 20×20, and the number of channels of the feature mapping is changed from 1 to 1024 after multiple downsampling operations. The convolution operation can be represented by Equation (7).

(7) $$Y_{s,t}=f(\sum_{m=1}^{3}\sum_{n=1}^{3}w_{m,n}\cdot x_{i+m,j+n}+b)$$

where $w_{m,n}$ represents the weight parameter of the convolution kernel in the *m*th row and the *n*th column, *b* represents the bias parameter, the convolution kernel’s width and height are 3 and 3, respectively, $x_{i+m,j+n}$ represents the pixels in the *i*th + *m*th row and *j*th + *n*th columns of the input image, and *i* and *j* represent the convolution kernel sliding over the input image by row *i* and columns *j*, respectively, *f*(⋅) represents the nonlinear activation function, and $Y_{s,t}$ represents the *s*th row and *t*th column of the output feature map of the convolution operation.

The nonlinear activation function used in the convolutional neural network is the Sigmoid function, which is defined as follows:

(8) $$f(x)=\frac{1}{1+e^{-x}}$$

Each module starts by doubling the size of the feature mapping and halving the number of channels through an up-convolution operation, and then the resulting feature mapping is connected to the feature mapping of the symmetric compressed path on the left, and the same convolution is performed on the combined feature mapping. Since this subsection first considers only the distinction between the foreground and background of the ISAR image satellite target, which is a binary semantic segmentation task, the feature mapping with a channel number of 2 is finally input.

### 3.2. Mask Matching Network

After having the semantic segmentation network model of the ISAR image, the binary mask of the ISAR image of the satellite target can be predicted. On this basis, it is further required to match the mask with the samples in the part labeled ISAR image dataset to predict the part labels of the ISAR image test samples and realize the ISAR image part recognition. Siamese networks for computing sample similarity have been successfully used in application scenarios such as face recognition, fingerprint recognition, and signature verification [39,40]; therefore, a mask matching model based on Siamese networks is designed in this study. Siamese networks, also known as biorthogonal neural networks, are coupled frameworks constructed using two artificial neural networks [41,42]. It takes two samples as input, represents them as feature vectors in a high-dimensional embedding space using a feature extraction module, and determines the similarity of the samples by comparing the distance between the sample feature vectors. With the rise of deep learning technology, current Siamese networks usually have a deep structure and can be composed of convolutional neural networks (CNN), recurrent neural networks (RNN), etc., in which the image similarity metric is generally used to extract its deep features using CNN.

**Figure 3:**
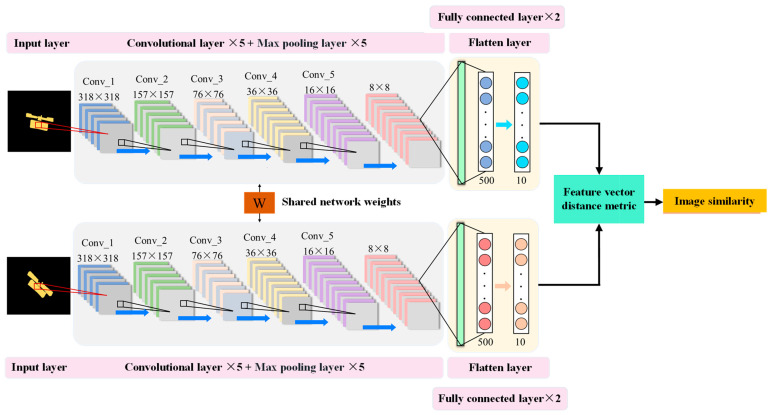
ISAR image binary mask matching Siamese network structure.

The ISAR images mask matching Siamese network model designed in this study is shown in Figure 3, which takes two satellites’ target ISAR image binary masks as input samples, size 320 × 320, and reduces the output feature mapping size to 8 × 8, through 5 convolutional operations with filter size 3 × 3, and 5 max pool operations with filter size 2 × 2, where the number of output channels of all convolutional operations is 64, i.e., the final size of the extracted feature map is 64 × 8 × 8. Then, the feature mapping is tiled, and the feature vector is changed to 500 and 10 dimensions using two fully connected layers. Finally, the Euclidean distance metric is used to calculate the similarity of the two input mask images. The Euclidean distance is calculated as shown in Equation (9):

(9) $$d(I_{1},I_{2})=\left\|G_{W}(I_{1})-G_{W}(I_{2})\right\|$$

where $G_{W}(I_{1})$ and $G_{W}(I_{2})$ denote the features extracted by the branch for the image of $I_{1}$ and $I_{2}$, respectively.

It should be emphasized that the feature extraction modules of the two input images have the same network structure and share the network parameters, so the similarity of the original images can be measured by using the distance of their feature vectors when the network models are the same.

When carrying out Siamese network model training, it is also necessary to design the appropriate loss function to make the network model parameters optimal in the process of reducing the loss function. In this study, the contrastive loss (CL) function is used [43], which can effectively handle the relationship between pairs of data in the Siamese network with the following expressions:

(10) $$CL=\frac{1}{2N}\sum_{n=1}^{N}\left[yd^{2}+\left(1-y\right)\max{\left(margin-d,0\right)}^{2}\right]$$

where $d={\left\|x_{1}-x_{2}\right\|}^{2}$ denotes the Euclidean distance of the eigenvectors of the two input samples, and y is the label of whether the two input samples match, where y=1 represents the sample similarity or match, and y=0 represents the mismatch, and margin is the set threshold (set to 1 in this study). When y=1, the loss function is only $\sum yd^{2}$, i.e., the original similar samples, and if their eigenvector Euclidean distance is larger, it means that the current model parameters are suboptimal and the loss needs to be increased; and when y=0, the loss function is $\sum \left(1-y\right)\max{\left(margin-d,0\right)}^{2}$, i.e., the samples are not similar, and the loss value will become larger if their eigenvector Euclidean distance is small instead, which is exactly in line with the requirements of Siamese network training. From the above, it can be found that this loss function is well suited to represent the degree of matching into samples and can also be used to train the feature extraction network model.

## 4. Experiment and Discussion

This section first prepares the satellite target ISAR images component annotation data for the network model training, then gives the evaluation metrics and loss functions of the network model, and finally presents the comparative analysis of the results with other classical images semantic segmentation models to highlight the advantages of the satellite target ISAR images component recognition method proposed in this study.

### 4.1. Satellite Target ISAR Image Component Labeling Data Preparation

Under the condition of having enough information about the satellite target, the designed automatic labeling technique is used to realize the satellite target ISAR image component labeling.

**Figure 4:**
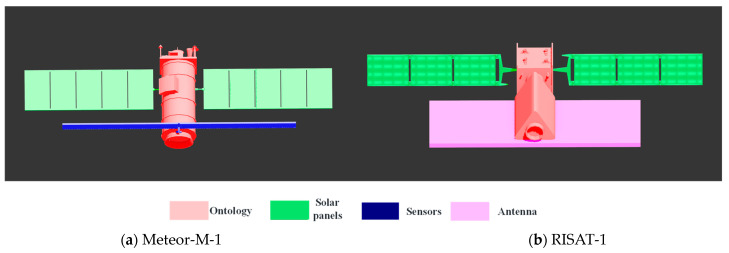
Satellite target parts labeled 3D model schematic.

Step 1: Satellite target 3D model component labeling. The 3Dmax-2020 software is used to be able to draw a reliable 3D model of the satellite target, based on which the software is further used to label the surface elements of each component of the satellite target, and the labeled 3D model file is converted and output into .3ds format. Figure 4 shows the component annotation of the 3D models of two satellites.

Step 2: Parsing the surface element information of each component of the 3D model. The OpenGL-based 3D model surface element analysis software is used for surface element analysis of the component labeled 3D model obtained from Step 1. The resolved file information not only contains the position coordinates and normal vector of each surface element in the satellite body coordinate system, but also the name of the component to which each surface element belongs.

Step 3: Determine the imaging parameters of the ISAR system and carry out the ISAR imaging simulation of the satellite target. Based on the surface element information of the satellite target obtained from Step 2 and the observable arc segment information of the satellite target obtained from STK11.3 software simulation, the MATLAB-based radar data processing and imaging application software is used to perform ISAR imaging of the satellite target, including specifying the imaging parameters of the ISAR system, dividing the observable arc segment into multiple imaging sub-apertures according to the imaging time, calculating the observable elements and its scattering coefficient, as well as generating ISAR broadband signals and satellite target echoes, and using the echo information for range-doppler imaging. Set the ISAR system imaging parameters to a signal carrier frequency of 14 GHz, a bandwidth of 2 GHz, and an imaging buildup time of 20 s.

Step 4: Determine the component names of the visible elements and the ISAR imaging coordinates. Based on Step 2 and Step 3, the component names of the visible elements of the satellite target 3D model can be determined; meanwhile, based on the distance between each face element in radial and azimuthal directions and the imaging center, as well as the distance unit length and doppler resolution of ISAR imaging, the coordinates of each face element in the ISAR image can be obtained, and then the pixel distribution of each component of the target in the ISAR image can be obtained. This enables the automatic labeling of satellite target ISAR image components.

**Figure 5:**
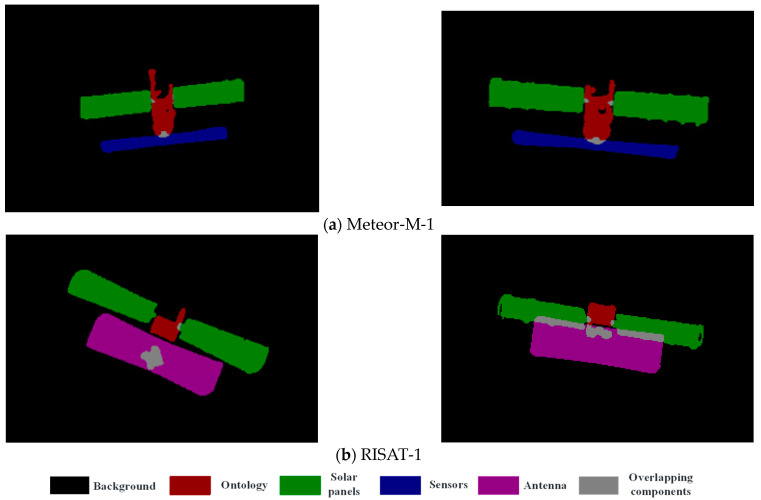
Typical ISAR images of the satellite targets identified by the components of this study.

In this study, the following two principles are followed in the selection of satellite targets: the overall size of the satellite is as large as possible to increase the number of pixels of the target in the ISAR image; the number of satellite components is not too many, and the distribution is not particularly complex. Therefore, two satellites, Meteor-M-1 and RISAT-1, are selected for component identification in this study. Their 3D model component annotations are shown in Figure 4, and Figure 5 shows their typical component-annotated ISAR images. The distribution is not particularly complex.

### 4.2. Evaluation Indicators and Network Model Training

#### 4.2.1. Evaluation Indicators

In image semantic segmentation tasks, the Jaccard metric is commonly used to evaluate the segmentation results. This metric, also known as the Intersection-over-Union (IoU) score, is the ratio of the intersection of two sets to the concatenation, and for semantic segmentation tasks, is expressed as the intersection ratio between the predicted label set and the true label set. In addition, individual pixel-based evaluation metrics are also commonly used to assess the quality of semantic segmentation results, such as Pixel Accuracy (PA), which is the ratio of the number of correctly predicted pixels to the total number of pixels in a semantically segmented image. For a multi-category image semantic segmentation task, the IoU scores and PA of all categories are generally averaged, i.e., Mean Intersection-Over-Union (MIoU) and Mean Pixel Accuracy (MPA). Assuming that the semantic segmentation image has a total of k+1 pixel categories, $p_{ij}$ denotes the number of pixels in category i that are predicted to be in category j. In this way, $p_{ii}$ denotes True Positives (TP), $p_{ij}$ and $p_{ji}$ denote False Positives (FP) and False Negatives (FN), respectively, at which point the above evaluation metrics are calculated as follows:

(11) $$\begin{array}{ll} PA=\frac{\sum_{i=0}^{k}p_{ii}}{\sum_{i=0}^{k}\sum_{j=0}^{k}p_{ij}}, & MPA=\frac{1}{k+1}\sum_{i=0}^{k}\frac{p_{ii}}{\sum_{j=0}^{k}p_{ij}}\\ IoU=\frac{\sum_{i=0}^{k}p_{ii}}{\sum_{i=0}^{k}\sum_{j=0}^{k}p_{ij}+\sum_{i=0}^{k}\sum_{j=0}^{k}p_{ji}-\sum_{i=0}^{k}p_{ii}}, & MIoU=\frac{1}{k+1}\sum_{i=0}^{k}\frac{p_{ii}}{\sum_{j=0}^{k}p_{ij}+\sum_{j=0}^{k}p_{ji}-p_{ii}} \end{array}$$

#### 4.2.2. Model Training

The ISAR image component identification method proposed in this study includes two network models, i.e., semantic segmentation and mask-matching network models, and data partitioning of satellite target component-labeled ISAR images is required before carrying out network training. In this study, all the part-labeled ISAR image samples of each satellite target are equally divided into two components, i.e., ISAR image test samples and ISAR images matching datasets with known component labels, where the ISAR image test samples are only used in the testing phase of the Siamese network model, and since the component labels of the samples in the ISAR images matching database are known, they can be used in the U-Net training and testing phases, as well as the training phase of the Siamese network. In this paper, we used a total of 1360 ISAR image samples, which were divided into ISAR image test samples and ISAR image matching datasets. Among them, a dataset of ISAR images with known labels was used, consisting of 680 samples. During the training and testing phases of the models, 476 training images and 204 testing images were employed for the U-Net model, while 680 training images and 680 testing images were used for the Siamese network. The training process of the U-Net semantic segmentation model and Siamese mask matching model is described in detail below.

**Figure 6:**
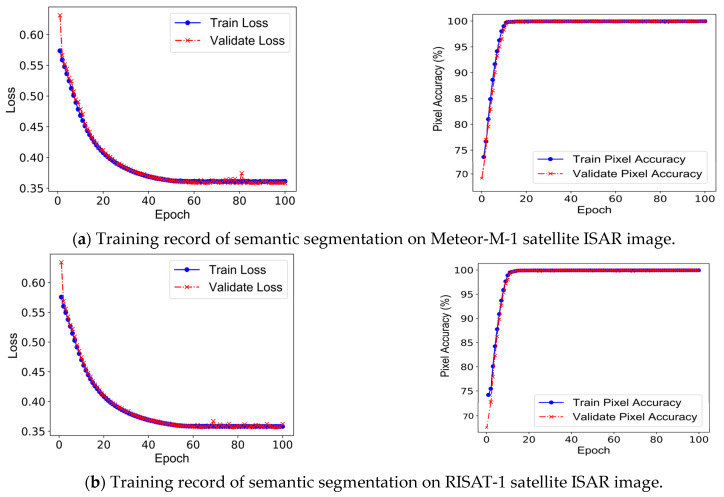
Training record of U-Net on satellite target ISAR image.

When conducting the training of the U-Net semantic segmentation network model, all ISAR image samples are divided into ISAR image training samples and test samples in the ratio of 7:3, and 30% of the ISAR image training samples are used as the validation set to adjust the hyperparameters of the network training and to determine the best network model parameters. Since the U-Net semantic segmentation network designed in this study only outputs the binary classification mask of the ISAR image, the loss function used is the Binary Cross-Entropy loss function, whose expression is shown in Equation (3), and the Adam optimization algorithm with an initial learning rate of 0.001 is used to reduce this loss. The training process of the U-Net network model with 100 Epochs is recorded in Figure 6, from which it can be seen that the U-Net model is trained on the ISAR images of two satellites. When training, both training loss and validation loss decrease with the increase of training times, and the pixel recognition accuracy of both training and validation samples gradually increases, and both loss values and recognition accuracy gradually level off at the later stage, which indicates that the U-Net model in this study is well trained.

**Figure 7:**
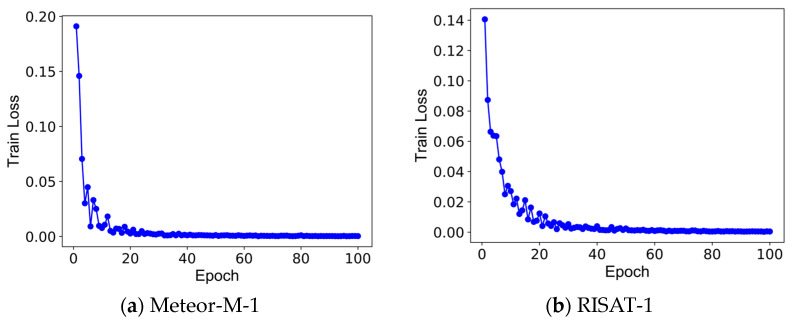
Training record of Siamese network on satellite target ISAR image mask.

During the training of the Siamese mask matching network model, similar to the U-Net semantic segmentation network model training, 30% of the ISAR image training samples were used as the validation set. Specifically, a total of 204 ISAR image training samples were selected as the validation set to fine-tune the hyperparameters of the S network training and determine the optimal network model parameters. When training the Siamese mask matching network model, an important task is to construct ISAR image binary mask matching sample pairs. In the pair construction process, the ISAR image binary mask sample is randomly rotated by a small angle and used as its own ISAR image binary mask matching sample; meanwhile, the binary mask sample of other ISAR images is randomly selected and randomly rotated by a larger angle and used as its ISAR image binary mask mismatch sample. At this point, pairs of ISAR image binary mask matched and unmatched samples are successfully constructed. The size of all ISAR image binary mask samples is 320 × 320, and the batch size is 8 at the time of sample collection. The loss function is the contrast loss (Equation (10)), and the Stochastic Gradient Descent (SGD) optimization algorithm with a learning rate of 0.001 and a momentum of 0.9 is used to reduce the training loss value to optimize the Siamese network model parameters. The training records of the Siamese network on the binary mask samples of Meteor-M-1 and RISAT-1 satellites are shown in Figure 7, from which it can be seen that after 100 Epochs of training, the value of the contrast loss function gradually decreases and is close to 0, which indicates that the Siamese network model is well trained and is not too hasty.

To prevent overfitting caused by limited datasets, we adopted two methods in this study. Firstly, we performed various data augmentation operations on the dataset, such as rotation, flipping, and folding. Through these operations, we extended the initial dataset. This data augmentation process helps to introduce more diversity and variability into the dataset, allowing the model to learn from a wider range of examples. Therefore, through data augmentation, we reduced the risk of overfitting and improved the model’s generalization ability for unseen examples. Secondly, satellites are generally composed of a body, solar panels, and sensors, so imaging them will generate similar ISAR satellite images. Based on this observation, we developed a specific ISAR image component automatic annotation technique to capture the features of such images. By training our model based on enhanced data and well-designed image processing techniques, we ensure that it has the necessary capabilities to process ISAR images.

### 4.3. Experimental Results and Comparative Analysis

After the training of the two network models is completed, the binary mask samples from the ISAR image test samples and the ISAR image matching database are input to the U-Net semantic segmentation network model and the Siamese mask matching network model, respectively, and the binary mask prediction results of the ISAR image test samples, as well as the ISAR image binary mask matching results and the predicted part labels can be obtained, respectively. The experimental results of the proposed method are presented below in terms of both the ISAR image binary semantic segmentation results and the ISAR image mask matching and component identification results.

#### 4.3.1. Experimental Results of ISAR Image Binary Semantic Segmentation

**Figure 8:**
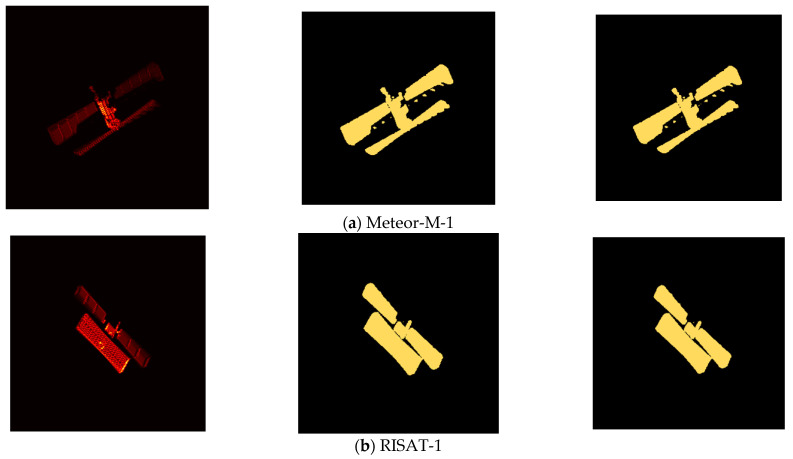
Example of binary semantic segmentation results of U-Net model for ISAR image test samples. Note: From left to right, the three columns of images are ISAR image, real mask, and prediction mask in order.

In the previous subsection, the ISAR image training samples and validation samples have been used as inputs to train the network for the ISAR image U-Net semantic segmentation model, and the parameter-optimized binary U-Net semantic segmentation model has been obtained. Here, the divided ISAR image test samples are used as inputs to test the performance of the above model for binary classification semantic segmentation. The binary semantic segmentation results of the U-Net model on the ISAR image test samples are given in Table 1, and a typical example is shown in Figure 8, from which it can be seen that for the ISAR image test samples of both Meteor-M-1 and RISAT-1 satellites, the U-Net model can achieve binary semantic segmentation very accurately, i.e., distinguish the ISAR image target and ISAR image backgrounds. Moreover, two semantic segmentation evaluation metrics, PA and IoU, are high.

#### 4.3.2. ISAR Image Mask Matching and Component Identification Results

The U-Net model can predict the binary masks of ISAR image test samples more accurately, which in turn provides a good basis for ISAR image mask matching and component identification. As shown in Figure 1, the binary masks of ISAR image test samples predicted by U-Net and the binary masks of the samples in the component-labeled ISAR image dataset are fed into the trained Siamese network at the same time, and the similarity between the binary masks of ISAR image test samples and the binary masks of all samples in the component-labeled ISAR image dataset can be obtained, and the most similar ISAR image component labels as the predicted part labels of the ISAR image test samples. Table 2 and Table 3 give the evaluation indexes of the proposed method for component identification of the ISAR image test samples of Meteor-M-1 and RISAT-1 satellites, respectively, and the examples of component identification results, from which it can be found that PAand MIoU of the proposed method in this study are relatively high and have validity and feasibility.

#### 4.3.3. Comparative Analysis and Discussion

**Figure 9:**
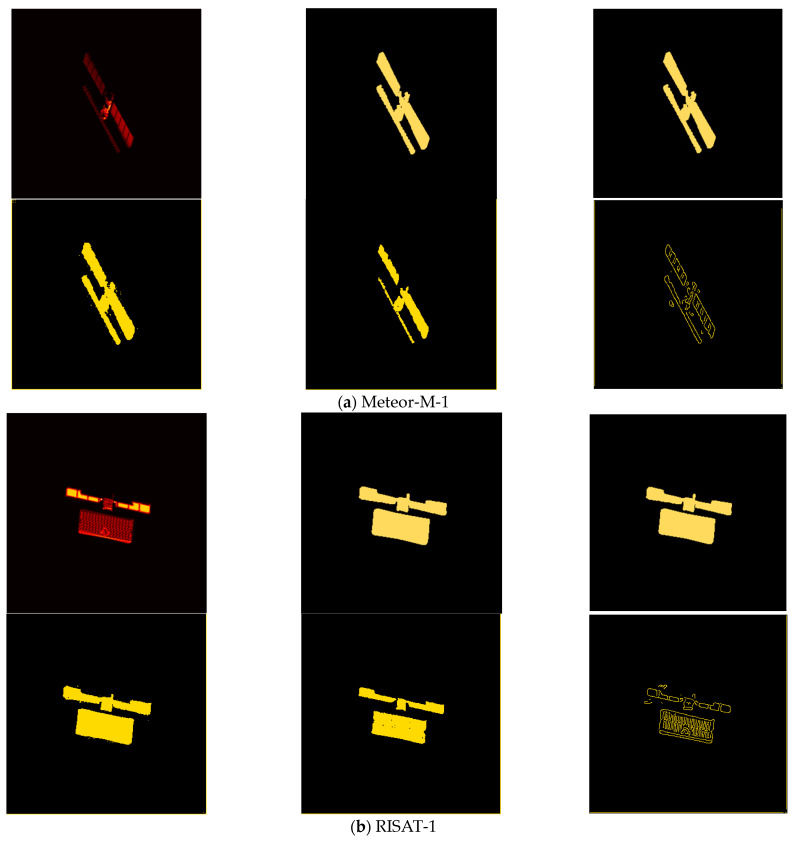
Example of binary semantic segmentation results with different methods for ISAR image test samples. Note: In the first row, from left to right, the three columns of images are ISAR image, real mask, and result of our method in order. In the second row, from left to right, the three columns of images are result of thresholding, result of K-mean clustering, and result of gradient-base in order.

To compare the superiority of the proposed semantic segmentation network, this subsection first uses traditional image segmentation methods to carry out a comparative study on the performance of binary semantic segmentation, including thresholding method, K-mean clustering, and gradient-based image segmentation. The performance of binary semantic segmentation with four methods for the ISAR images of Meteor-M-1 and RISAT-1 satellites is given in Table 4. Figure 9 shows an example of the binary semantic segmentation results for one of the ISAR images. The comparison of the experimental results shows that the proposed method has the best performance in binary semantic segmentation of satellite ISAR images, and the binary semantic segmentation results are closest to the real results.

**Figure 10:**
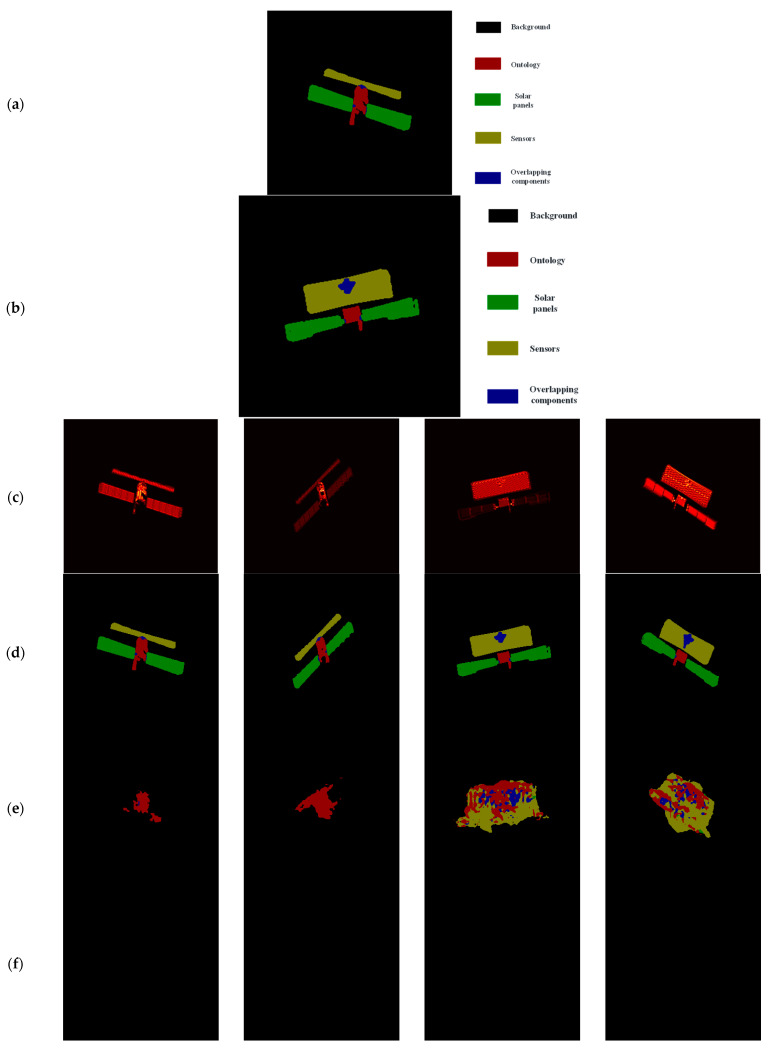

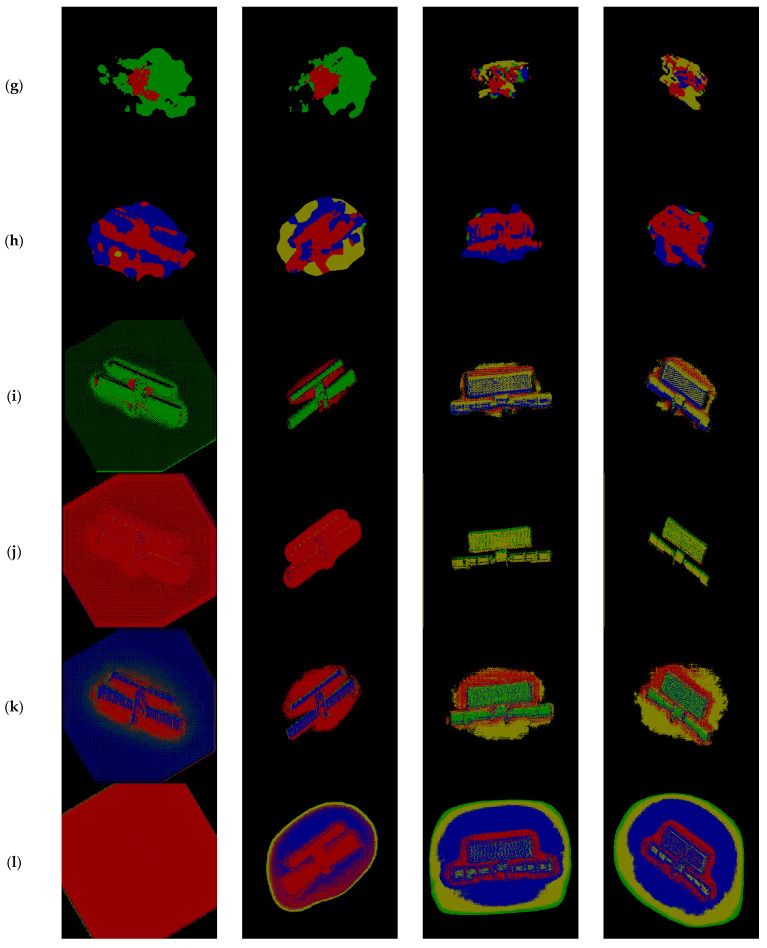

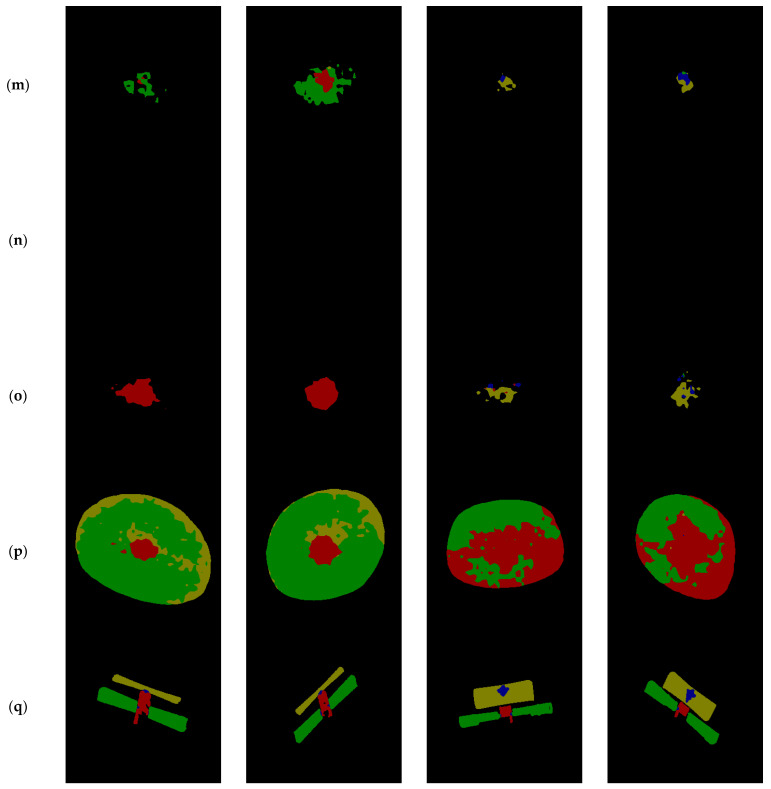
Example of ISAR images component recognition results for different methods, (**a**) Meteor-M-1 real label legend; (**b**) RISAT-1 real label legend; (**c**) ISAR image; (**d**) real label; (**e**) DeepLab v3+ and Cross-Entropy; (**f**) DeepLab v3+ and Dice; (**g**) DeepLab v3+ and Focal; (**h**) DeepLab v3+ and Lovasz-Softmax; (**i**) U-Net and Cross-Entropy;(**j**) U-Net and Dice; (**k**) U-Net and Focal; (**l**) U-Net and Lovasz-Softmax; (**m**) PSPNet and Cross-Entropy; (**n**) PSPNet and Dice; (**o**) PSPNet and Focal; (**p**) PSPNet and Lovasz-Softmax; (**q**) our method.

To further illustrate the advantages of the proposed method, In this subsection, three current classical image semantic segmentation neural network models, DeepLab v3+ [44], U-Net [28], and PSPNet [35], are used to carry out a comparative study on the performance of satellite target ISAR image component recognition; for each semantic segmentation network model, the initial learning rate of 0.001 and momentum of 0.9 stochastic gradient descent (SGD) algorithm to optimize the loss functions in Section 4.2.1, including cross-entropy loss, Dice loss, focal loss, and Lovasz-Softmax loss. The average performance of the four methods for component recognition under different loss function optimizations for all Meteor-M-1 and RISAT-1 satellite ISAR images in the test set is given in Table 5, and Figure 10 shows the component recognition results for one of the typical ISAR images selected in the test set. By comparing the experimental results, it can be seen that no matter what loss functions are used to optimize the above three classical semantic segmentation network models, DeepLab v3+ [44], U-Net [28], and PSPNet [35], their semantic segmentation performance for satellite target ISAR images is not satisfactory, while the method proposed in this study can identify the components of satellite target ISAR images more accurately and has a relative advantage.

Compared with the classical image semantic segmentation model, the experimental results of this study achieve higher IoU scores, which proves that the proposed method has obvious advantages in ISAR image components recognition. Meanwhile, subsequent scholars can realize more accurate ISAR image part recognition by improving the U-Net and Siamese networks or designing more appropriate semantic segmentation models and matching models. An important point is that the results of this study provide additions to the existing literature in the field of space engineering. Through our study, we propose a semantic segmentation and mask-matching-based ISAR image part recognition method, which employs the designed U-Net and Siamese networks to implement ISAR image binary semantic segmentation and binary mask matching, respectively, and use the mask matching results to predict the ISAR image part labels. The experimental results confirm the effectiveness and feasibility of this method, which provides new perspectives and ideas for researchers in the field of space engineering. The proposed method can also increase the number of samples in the satellite target component annotation ISAR image dataset, such as using ISAR image rotation, scaling, and flipping to enhance the data, to achieve the optimal matching between the ISAR image test samples and the dataset samples, which can further improve the ISAR image component recognition performance of the proposed method and can be successfully applied to the satellite target ISAR image component identification task.

We recognize that our method may have some limitations when multiple satellite target components need to be identified simultaneously, especially when dealing with satellite targets with similar form factors and considering the possible negative impact of ISAR imaging parameters. In such cases, the recognition accuracy of our method may be degraded. At this point, it is necessary to improve the above U-Net and Siamese networks or design more appropriate semantic segmentation models and matching models to achieve more accurate ISAR component recognition. Meanwhile, the recognition accuracy of this research method also relies on the sample completeness of the satellite target component-labeled ISAR image dataset.

## 5. Conclusions

In this paper, an automatic ISAR image component labeling method is designed to obtain satellite target component labeling ISAR image samples and datasets accurately and efficiently. Based on this, a semantic segmentation and mask-matching-based ISAR image component identification method is proposed, and the designed U-Net and Siamese networks are used to implement ISAR image binary semantic segmentation and binary mask matching, respectively, and the mask matching results are used to predict the component labels of ISAR images. Based on several classical semantic segmentation network models and loss functions, experiments on multiclassification semantic segmentation of ISAR images are conducted, and their experimental results are compared and analyzed with the proposed method in this study, and the results confirm that the proposed method has significant advantages.

## Figures and Tables

**Table 1:** Binary semantic segmentation results of U-Net for ISAR-like test samples.

Satellite Targets	PA (%)	IoU
Meteor-M-1	99.94%	0.9961
RISAT-1	99.94%	0.9974

**Table 2:** Component recognition index results of satellite target ISAR images.

Satellite Targets	PA (%)	IoU_cls0_	IoU_cls1_	IoU_cls2_	IoU_cls3_	IoU_cls4_	MIoU
Meteor-M-1	96.88	0.9731	0.7139	0.7211	0.5724	0.4293	0.6820
RISAT-1	97.11	0.9811	0.7519	0.6541	0.7625	0.5788	0.7457

Note: IoU_cls0_ denotes the Intersection-over-Union (IoU) of background, IoU_cls1_ denotes the IoU of ontology, IoU_cls2_ denotes the IoU of solar panels, IoU_cls3_ denotes the IoU of sensors, and IoU_cls4_ denotes the IoU of overlapping components. MIoU denotes Mean Intersection-Over-Union of the whole image.

**Table 3:** Example of satellite target ISAR image component identification results.

	ISAR Image	Binarization Mask	Multiclassification Masks
**Real situation**	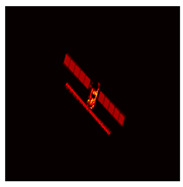	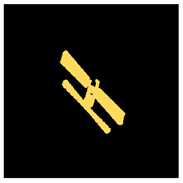	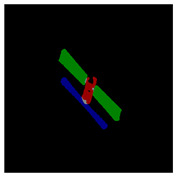
**Matching**	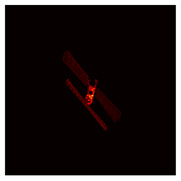	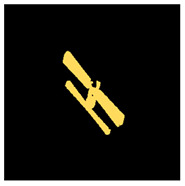	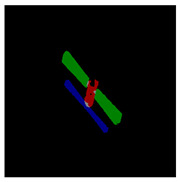
(**a**) Meteor-M-1			
**Real situation**	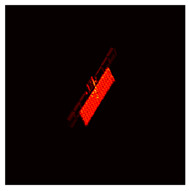	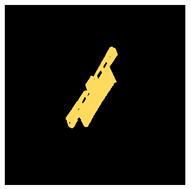	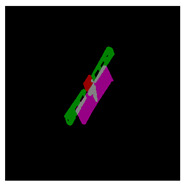
**Matching**	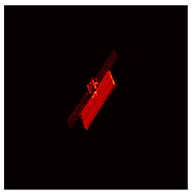	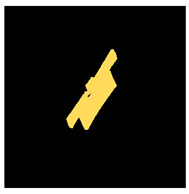	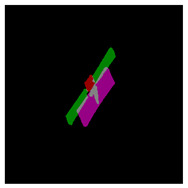
(**b**) RISAT-1			

**Table 4:** The performance of ISAR image binary semantic segmentation for satellite targets with different methods.

Satellite Targets	Method	PA (%)	IoU	Satellite Targets	Method	PA (%)	IoU
Meteor-M-1	Thresholding	97.65	0.9632	RISAT-1	Thresholding	99.01	0.9812
	K-mean clustering	93.81	0.8596		K-mean clustering	96.37	0.9474
	Gradient-base	88.05	0.6024		Gradient-base	90.14	0.7352
	**Our Method**	99.92	0.9951		**Our Method**	99.95	0.9981

**Table 5:** The average performance of ISAR image component identification for satellite targets with different methods.

Satellite Targets	Network Model	Loss Function	PA (%)	IoU_cls0_	IoU_cls1_	IoU_cls2_	IoU_cls3_	IoU_cls4_	MIoU
Meteor-M-1	DeepLab v3+	Cross-Entropy	88.98	0.922	0.286	0.0	0.0	0.003	0.242
		Dice	88.07	0.918	0.0	0.0	0.0	0.0	0.184
		Focal	89.03	0.922	0.312	0.0	0.0	0.007	0.248
		Lovasz-Softmax	80.27	0.744	0.343	0.100	0.061	0.038	0.257
	U-Net	Cross-Entropy	88.81	0.921	0.231	0.0	0.0	0.003	0.231
		Dice	88.91	0.922	0.252	0.0	0.0	0.007	0.236
		Focal	88.81	0.921	0.227	0.0	0.0	0.007	0.231
		Lovasz-Softmax	80.59	0.754	0.224	0.091	0.065	0.004	0.235
	PSPNet	Cross-Entropy	89.05	0.922	0.313	0.003	0.0	0.003	0.248
		Dice	88.89	0.922	0.242	0.0	0.0	0.0	0.233
		Focal	89.00	0.922	0.298	0.0	0.0	0.002	0.244
		Lovasz-Softmax	81.16	0.757	0.347	0.113	0.066	0.052	0.267
	**Our Method**	**Binary Cross-Entropy** **and Contrastive Loss**	**96.88**	**0.973**	**0.714**	**0.721**	**0.572**	**0.429**	**0.682**
RISAT-1	DeepLab v3+	Cross-Entropy	88.97	0.937	0.081	0.007	0.236	0.049	0.262
		Dice	86.01	0.921	0.0	0.0	0.0	0.0	0.184
		Focal	89.13	0.938	0.072	0.006	0.256	0.047	0.264
		Lovasz-Softmax	84.10	0.830	0.169	0.077	0.229	0.089	0.279
	U-Net	Cross-Entropy	89.37	0.938	0.098	0.004	0.290	0.038	0.274
		Dice	85.88	0.918	0.001	0.002	0.0	0.01	0.186
		Focal	89.23	0.937	0.110	0.005	0.272	0.036	0.272
		Lovasz-Softmax	83.24	0.826	0.146	0.059	0.161	0.076	0.253
	PSPNet	Cross-Entropy	89.32	0.938	0.113	0.010	0.272	0.062	0.283
		Dice	86.45	0.926	0.147	0.0	0.002	0.0	0.215
		Focal	89.23	0.937	0.125	0.009	0.265	0.007	0.281
		Lovasz-Softmax	83.47	0.821	0.173	0.075	0.210	0.105	0.277
	**Our Method**	**Binary Cross-Entropy** **and Contrastive Loss**	**97.11**	**0.981**	**0.752**	**0.654**	**0.763**	**0.579**	**0.746**

Note: IoU_cls0_ denotes the Intersection-over-Union (IoU) of background, IoU_cls1_ denotes the IoU of ontology, IoU_cls2_ denotes the IoU of solar panels, IoU_cls3_ denotes the IoU of sensors, and IoU_cls4_ denotes the IoU of overlapping components. MIoU denotes Mean Intersection-Over-Union of the whole image.

## Data Availability

Not applicable.
